# Phenotypic alterations in immune cells and their association with toxicities in postoperative endometrial cancer patients undergoing brachytherapy

**DOI:** 10.3389/fonc.2025.1578394

**Published:** 2025-07-16

**Authors:** Naiping Sun, Jianting Liu, Zhulin Dong, Ruixin Suo, Junli Ren

**Affiliations:** ^1^ Department of Radiotherapy, Shanxi Province Cancer Hospital, Taiyuan, China; ^2^ Department of Preventive Care, Shanxi province Cancer Hospital, Taiyuan, China

**Keywords:** phenotype, immune cells, brachytherapy, endometrial cancer, toxicities

## Abstract

**Objective:**

This study aims to investigate the phenotypic alterations in peripheral blood immune cells, especially natural killer (NK) cells, natural killer T (NKT) cells, total B cells, total T cells and their subsets, in postoperative patients with intermediate-risk endometrial cancer undergoing brachytherapy, and explores their association with adverse reactions.

**Methods:**

A cohort of ninety-two patients, who received brachytherapy (5.0 Gy per fraction, 6 fractions) at Shanxi Cancer Hospital from January 2022 to November 2024, was included in this study. Immune cell subsets, including CD45^+^CD3^-^CD16^+^CD56^+^ NK cells, CD3^+^CD56^+^ NKT cells, CD45^+^CD3^-^CD19^+^ B cells, CD3^+^CD8^+^ T cells, CD3^+^CD4^+^ CD4^+^ helper T cells, CD3^+^CD8^+^ Cytotoxic T cells, CD45^+^CD4^-^CD25^HI^CD127^LO^ Regulatory T cells, were quantified using flow cytometry pre-, during, and post- treatment. Various statistical methods were applied to analyze these phenotypic changes and the chi-square test was employed to explore their association with toxicities.

**Results:**

Compared to pre-treatment levels, NK cells and NKT cells increased significantly during and post-treatment (P<0.001, P=0.003), while B cells decreased significantly(P<0.001). No notable alterations were observed in the counts of total T cells and their subsets throughout the treatment process (P>0.05). Brachytherapy caused mild side effects, with no toxicities of grade II or higher observed. The incidence of acute radiation proctitis was significantly higher in the group with elevated NK cell levels compared to the non-elevated group (31.9% vs. 8.7%, P=0.028). The incidence of radiation proctitis, cystitis, and vaginitis did not differ significantly among the other groups (P>0.05).

**Conclusion:**

Brachytherapy enhances innate immune activation through the upregulation of NK/NKT cells, potentially correlating with the development of acute proctitis. These findings highlight immune modulation as a prospective biomarker for managing radiotherapy-induced toxicity.

## 1 Introduction

The incidence rate of endometrial cancer ranks sixth among malignant tumors in women (1) and has been increasing, particularly among younger women, over the past two decades, with the improvement of living standards (1). Surgical intervention remains the primary treatment modality for endometrial cancer (2). However, local recurrence is a significant cause of treatment failure, with an overall postoperative recurrence rate of approximately 13% (3). Prophylactic irradiation of potential subclinical lesion areas post-surgery is a crucial strategy for reducing recurrence. According to the European Society for Medical Oncology (ESMO) guidelines for postoperative adjuvant treatment of endometrial cancer, brachytherapy is recommended for patients with medium, medium-high, and high-risk profiles (4). Specifically, for patients with medium-risk profiles, brachytherapy is advised as a standalone treatment (4). The National Comprehensive Cancer Network (NCCN) guidelines suggest brachytherapy at a dose of 7.0 Gy/fx 3 fractions or 5.0 Gy/fx 6 fractions (5). The underlying mechanism of tumor development is attributed to immune escape (6). By increasing the infiltration of CD8+ and CD4+ T lymphocytes, radiotherapy enhances the ability to identify and eradicate tumor cells, strengthening the anti-tumor immune effect and inhibiting immune escape (7). While radiation’s impact on immune escape mechanisms is recognized, there is limited data on how brachytherapy alters peripheral immune cell phenotypes and their association with toxicity. Prior studies have focused on the immunosuppressive effects of external beam radiotherapy, however, the localized high-dose radiation characteristic of brachytherapy may uniquely modulate innate immunity. Given the anatomical proximity of the vaginal stump to adjacent organs, such as bladder and rectum, the occurrence of radiation proctitis and, cystitis and vaginitis is unavoidable. Although a correlation between radiation injury and radiation dose has been established (8), radiation damage differs even with the same radiation dose and there is limited research on the potential correlation between radiation injury and phenotypic changes in immune cells induced by brachytherapy. This study addresses these gaps by analyzing the phenotypic alterations of immune cells in patients with endometrial cancer undergoing postoperative brachytherapy before, during, and after brachytherapy, as well as their clinical implications.

## 2 Materials and methods

### 2.1 Patient eligibility

Ninety-two patients diagnosed with endometrial cancer, who underwent staging surgery followed by intracavitary brachytherapy at Shanxi Cancer Hospital between January 2022 and June 2024, were selected for this study.

The inclusion criteria: (1)Histopathological confirmed endometrial cancer; (2) Classification into the medium-risk group, defined as: stage IA with low-grade tumors exhibiting focal Lymph-Vascular Space Invasion(LVSI) (1 to 3 vessels are involved) or patients aged 60 years or older; stage IA with high-grade tumors; Unique pathological type (Serous adenocarcinoma, Clear cell adenocarcinoma, Undifferentiated carcinoma, Carcinosarcoma, Mixed carcinoma) in stage IA without infiltration of the muscle layer; stage IB with low-grade tumors, based on the FIGO 2009 surgical pathology staging system and the ESGO-ESTRO-ESP guidelines for postoperative adjuvant therapy in endometrial cancer management (9); (3) A Karnofsky Performance Status score of at least 70; (4) Adults aged 20–80 years old; (5) Complete and reliable clinical information and follow-up data.

Exclusion criteria: (1) Exposure to external radiation, chemotherapy, immunotherapy, targeted therapy, or any other anti-tumor treatments, as well as leukocyte-boosting therapies before and during brachytherapy; (2) Combined with other tumors. Informed consent for brachytherapy was obtained from all patients prior to treatment.

### 2.2 Brachytherapy

Brachytherapy is initiated 4–8 weeks after surgery. The patient was placed in the lithotomy position, disinfected, and then a urinary catheter was inserted. A gynecological exam was conducted with a gynecological dilator, and local anesthesia was applied using Obukaine gel. An appropriately sized oval applicator was then placed into the vaginal stump. Gauze was used to pack the vagina to increase the distance of the source position to the bladder and rectum and to fix the applicator, after which the gynaecological dilator was removed. Once the applicator was placed, 150 ml of 0.9% NaCl solution was infused into the bladder. CT localization was conducted using a SOMATOM Confidence large aperture CT simulator from Siemens, Germany, with a slice thickness of 2 mm. The scanning range covered the entire pelvic cavity. The CT images were then transferred to Oncenta Brachay system for contouring and planning. The high-risk clinical target area (HR-CTV) is defineated according to the recommendations of the ESMO-ESGO-ESTRO consensus (10). The outer contours of the rectum, sigmoid colon, small intestine, and bladder were outlined as organs at risk (OAR). A total of six fractions of 5Gy were performed. Make sure that the therapeutic dose covers at least 90% of the target volume. Prior to treatment, the bladder volume was assessed using color Doppler ultrasound to ensure consistency with the volume recorded during localization. Radiation source is Iridium-192(^192^Ir) and the ^192^Ir high-dose rate brachytherapy is conducted using the Elekta afterloading therapy machine.

### 2.3 Flow cytometric Immunophenotyping

The research investigates the phenotypic changes of immune cells in the peripheral blood of patients before, during, and after brachytherapy. The time intervals are defined as follows: The pre-treatment phase (before brachytherapy) defined as the period from 0 to 24 hours prior to the initiation of brachytherapy; the treatment phase (during brachytherapy) covers the time span from the end of the third fraction to the start of the fourth fraction of brachytherapy; the post-treatment phase (after brachytherapy) defined as the day after brachytherapy ends.

Flow cytometric Immunophenotyping performed on fresh peripheral blood. Collect 2 ml of venous blood from patients and extract serum following a minimum of 8 hours of fasting in the morning. BD FACSCanto™ II flow cytometry was used for immunophenotyping. Cells were stained with fluorochrome-conjugated antibodies (BD Biosciences) against CD3, CD4, CD8, CD16, CD56, CD19, CD45, CD25, and CD127. Gating strategies adhered to ISAC guidelines and was validated with healthy donor controls. Data were collected on a BD FACSCanto™ II and analyzed using FlowJo v10. The antibody group used is as follows: total T cells: CD45+CD3+, CD4^+^ helper T cells:CD3^+^CD4^+^, Cytotoxic T cells:CD3^+^CD8^+^, Natural Killer Cells(NK Cells): CD45^+^CD3^-^CD16^+^CD56^+^, Natural killer T cells (NKT cells): CD3^+^CD56^+^, Regulatory T cells: CD45^+^CD4^-^CD25^HI^CD127^LO^, Total B cells: CD45^+^CD3^-^CD19^+^.

### 2.4 Follow up and assessment of toxicities

Follow-up evaluations were scheduled every 3 months for the first 2 years after brachytherapy, every 6 months subsequently. The follow-up programs included urine routine, stool routine, gynecological examinations, tumor marker (CEA, CA125, CA19-9), and enhanced CT scans of the chest, abdomen and pelvis. The primary adverse reaction evaluated were acute and chronic radiation cystitis, proctitis, and vaginitis, assessed according to the Common Terminology Criteria for Adverse Events (CTCAE 5.0) (11), established by the National Cancer Institute in the United States. Acute adverse reactions are defined as those occurring within 90 days after brachytherapy, while chronic adverse reactions are those occurring beyond 90 days.

### 2.5 Statistical analysis

Statistical analysis was conducted using SPSS 27.0 software. Count data is expressed as a percentage (%). Quantitative data following a normal distribution were presented as mean ± standard deviation. Comparisons across multiple groups were conducted using one-way repeated measures analysis and pairwise comparisons were conducted using paired t-tests for data following a normal distribution. Quantitative data that did not follow a normal distribution were represented by the Median (Q25, Q75), and pairwise comparisons were performed using the paired rank sum test. A P-value of less than 0.05 (P<0.05) was considered statistically significant. The chi-square test was used to evaluate differences between immunophenotype and toxicity, with a P-value under 0.05 (P<0.05) indicating statistical significance. The baseline clinical characteristics of the different groups of patient were compared using an independent sample t-test.

### 2.6 Ethics

This study protocol received approval from the institutional review board (Ethics Batch Number: KY2024183). All treatment must have informed consent.

## 3 Result

### 3.1 General information

In this study, 92 patients with moderate-risk endometrial cancer who had surgery were included, with a median age of 55 years(ranging from 30 to 73 years), and a median tumor size of 4cm (ranging from 1 to 10 cm). All patients received brachytherapy at a prescribed dose of 5.0 Gy/fx 6 fractions, twice a week and a dose of 5Gy was normalized to a depth of 5 mm below the vaginal mucosa (**Table 1**).

**Table 1:** The baseline clinical characteristics of patients.

Characteristics	n(%)
Median age (years, range)	55 (30-73)
Median tumor Size(cm,range)	4 (1-10)
Tumor staging(FIGO)	
IA with low-grade	47 (51.1%)
IA with high-grade	15 (16.3%)
IB with low-grade	30 (32.6)
Histological type	
Adenocarcinoma	88 (95.7%)
Clear cell carcinoma	3 (3.3%)
Serous carcinoma	1 (1.1%)

### 3.2 Alterations in phenotype of immune cells in the peripheral blood at different time points

**Figure 1:**
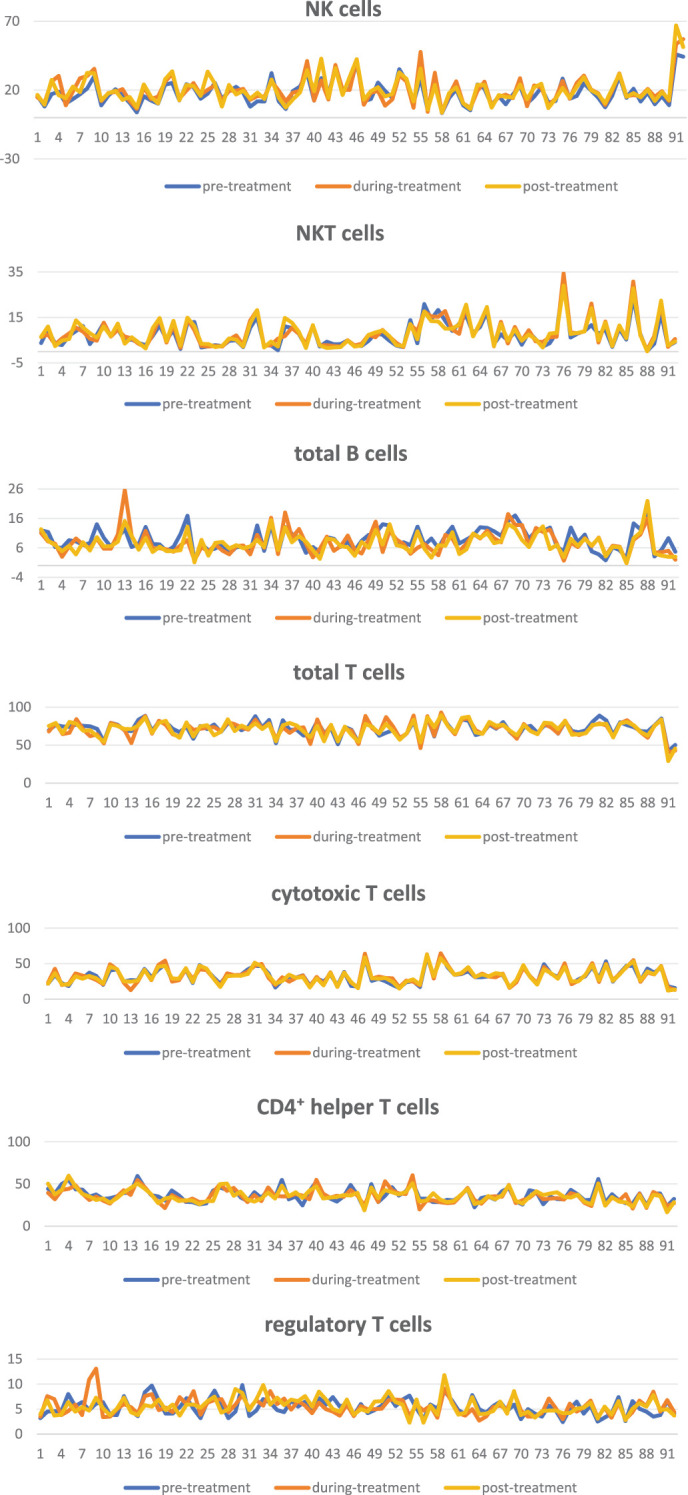
Line graph of alterations in phenotype of immune cells during treatment.

There was a significant increase in the levels of NK cells and NKT cells during and post-treatment compared to that of pre-treatment. (p<0.001 for both comparisons of NK cells, p=0.003 for both comparisons of NKT cells). Conversely, the levels of total B cells decreased significantly during and post-treatment compared to pre–treatment levels (p<0.001,p<0.001, **Table 2**, **Figure 1**).

**Table 2:** Comparison of the levels of NKcells,NKTcells and total B cells at different time.

Items and p-value	NK cells Median (Q25, Q75)	NKT cells Median (Q25, Q75)	Total B cells Median (Q25, Q75)
Pre-treatment(%)	16.15 (11.90, 22.55)	6.45 (3.20, 10.25)	8.10 (6.225, 11.775)
During treatment(%)	19.10 (13.27, 26.075)	6.65 (3.45, 10.725)	7.10 (5.30, 9.95)
Post-treatment(%)	17.95 (13.65, 23.95)	6.80 (3.325, 11.75)	6.90 (5.325, 9.375)
Z _pre VS dur_	-4.186	-3.016	-3.299
*P*-value _pre VS dur_	<0.001*	0.003*	<0.001*
Z _pre VS post_	-4.231	-3.009	-4.236
*P*-value _bef VS post_	<0.001*	0.003*	<0.001*
Z _durVS post_	-0.115	-0.069	-1.163
*P*-value _durVS post_	0.909	0.945	0.245

*P<0.05. pre, pre-treatment; dur, during treatment; post, post-treatment.

There were no significant alterations in the levels of NK cells, NKT cells, and total B cells between treatment and post-treatment (P > 0.05, **Table 2**, **Figure 1**). No notable alterations were observed in the levels of total T cells and T cell subsets, including auxiliary, cytotoxic, and regulatory T cells, throughout the treatment process (P > 0.05, **Table 3**, **Figure 1**).

**Table 3:** Comparison of T cells and its subsets at different time.

Items and p-value	Total T cells Median (Q25, Q75)	Cytotoxic T cells Median (Q25, Q75)	CD4^+^ helper T cells Mean ± SD	Regulatory T cells Median (Q25, Q75)
Pre-treatment(%)	73.05 (66.35, 78.15)	31.60(24.725, 41.325)	36.60 ± 8.21	5.10 (4.10, 6.50)
During treatment(%)	71.95 (64.57, 78.12)	31.20 (24.60, 41.325)	35.52 ± 8.04	5.35 (4.25, 6.70)
Post-treatment(%)	74.1 (65.425, 78.225)	31.85 (25.225, 40.625)	35.80 ± 7.818	5.30 (4.30, 6.6)
Z/F^*^_pre VS dur_	-1.869	-0.666	2.227	-0.614
*P*-value _pre VS dur_	0.062	0.505	0.111	0.539
Z _pre VS post_	-0.888	-0.201		-1.083
*P*-value _bef VS post_	0.375	0.841		0.279
Z _durVS post_	-0.552	-1.009		-0.745
*P*-value _durVS post_	0.581	0.313		0.456

Data following a normal distribution is analyzed using ANOVA, yielding an F statistic; data not following a normal distribution, comparisons between two groups were analyzed using a paired rank sum test with Z as the statistical value.

### 3.3 Correlation between the phenotypic Alterations of immune cells and the occurrence and severity of acute and chronic radiation cystitis, proctitis and vaginitis

By the end of the follow-up, no patients suffered from grade II or higher acute or chronic toxicities like radiation cystitis, proctitis, or vaginitis. When there are no side effects, it is termed 0-grade toxicities.There were 24 cases (26.1%) of grade I acute radiation proctitis and 19 cases (20.7%) of chronic radiation proctitis. Additionally, 14 cases (15.2%) of grade I acute radiation cystitis and 10 cases (10.9%) of chronic radiation cystitis were observed. Furthermore, 18 cases (19.6%) of grade I acute radiation vaginitis and 25 cases (27.2%) of chronic radiation vaginitis were reported.

By the end of brachytherapy, there were notable alterations in NK cells, NKT cells, and total B cells compared to pre-treatment. As a result, patients were divided into groups such as NK cell elevation and non-elevation, NKT cell elevation and non-elevation, and total B cell reduction and non-reduction. The incidence of acute radiation proctitis in the NK cell elevation group was 31.9%, whereas it was 8.7% in the NK cell non-elevation group, with the difference being statistically significant (P=0.028). The incidence of chronic radiation proctitis in the NK cell elevation group was 26.1%, compared to 4.3% in the NK cell non-elevation group, however, this difference did not reach statistical significance (P=0.053, refer to **Table 3**). The incidence of radiation cystitisand vaginitis did not differ significantly between the NK cell elevation group and the non-elevation group (P> 0.05, see **Tables 4**, **5**). Additionally, there were no statistically significant differences in the occurrence of acute and chronic radiation proctitis, cystitis, and vaginitis between the NKT cell elevation and non-elevation groups, between total B cell reduction and non-reduction groups (P> 0.05, see **Tables 6**, **4**, **5**).

**Table 4:** Correlation between the phenotypic changes of immune cells and the occurrence of radiation cystitis.

Items	0-dgradeegree acute radiation cystitis n(%)	2-grade 3-degreeacute radiatio cystitis n(%)	incidence(%)	*χ*2	*P*-value	0-grade chronic radiation cystitis n(%)	1-grade chronic radiation cystitis n(%)	incidence(%)	*χ*2	*P*-value
NK cell			1.797	0.180				0.598	0.439
ele	56 (60.9%)	13 (14.1%)	18.8%			60 (65.2%)	9 (9.8%)	13.0%		
non-ele	22 (23.9%)	1 (1.1%)	4.3%			22 (23.9%)	1 (1.1%)	4.3%		
NKT cell			0.382	0.536				2.214	0.145
ele	49 (53.3%)	10 (10.9%)	16.9%			50 (54.3%)	9 (9.8%)	15.3%		
non-ele	29 (31.5%)	4 (4.3%)	12.1%			32 (34.8%)	1 (1.1%)	3%		
total B cell			0.435	0.510				0	1
red	51 (55.4%)	11 (12.0%)	17.7%			55 (59.8%)	7 (7.6%)	11.3%		
non-red	27 (29.3%)	3 (3.3%)	10%			27 (29.3%)	3 (3.3%)	10%		
sum	78 (84.8%)	14 (15.2%)				82 (89.1%)	10 (10.9%)			

**Table 5:** Correlation between the phenotypic changes of immune cells and the occurrence of radiation vaginitis.

Items	0-grade acute radiation vaginitis n(%)	1-grade acute radiatio vaginitis n(%)	incidence(%)	*χ*2	*P*-value	0-grade chronic radiation vaginitis n(%)	1-grade chronic radiation vaginitis n(%)	Incidence(%)	*χ*2	*P*-value
NK cell				1.473	0.225				0.458	0.499
ele	53 (57.6%)	16 (17.4%)	23.2%			49 (53.3%)	20(21.7%)	29.0%		
non-ele	21 (22.8%)	2(2.2%)	8.7%			18 (19.6%)	5 (5.4%)	21.7%		
NKT cell				0.063	0.802				0.924	0.336
ele	47 (51.1%)	12 (13.0%)	20.3%			41(44.6%)	18 (19.6%)	30.5%		
non-ele	27 (29.3%)	6(6.5%)	20%			26 (28.3%)	7 (7.6%)	21.2%		
total B cell				1.099	0.295				0.180	0.672
red	48 (52.2%)	14 (15.2%)	22.6%			46 (50%)	16 (17.4%)	25.8%		
non-red	26 (28.3%)	4 (4.3%)	13.3%			21(22.8%)	9 (9.8%)	30%		
sum	74 (80.4%)	18(19.6%)				67 (72.8%)	25 (27.2%)			

**Table 6:** Correlation between the phenotypic changes of immune cells and the occurrence of radiation proctitis.

Items	0-grade acute radiation proctitis n(%)	1-grade acute radiation proctitis n(%)	Incidence(%)	*χ*2	*P*-value	0-grade chronic radiation proctitis n(%)	1-grade chronic radiation proctitis n(%)	Incidence(%)	*χ*2	*P*-value
NK cell				4.810	0.028*				3.737	0.053
ele	47 (51.1%)	22 (23.9%)	31.9%			51 (55.4%)	18(19.6%)	26.1%		
non-ele	21 (22.8%)	2 (2.2%)	8.7%			22 (23.9%)	1(1.1%)	4.3%		
NKT cell				1.668	0.197				0.950	0.330
ele	41 (44.6%)	18 (19.6%)	30.5%			45 (48.9%)	14 (15.2%)	23.7%		
non-ele	27 (29.3%)	6 (6.5%)	18.2%			28 (30.4%)	5 (5.4%)	15.2%		
total B cell				0.175	0.676				0.012	0.914
red	45(48.9%)	17 (18.5%)	27.4%			49 (53.3%)	13 (14.1%)	21.0%		
non-red	23 (25%)	7(7.6%)	23.3%			24 (26.1%)	6 (6.5%)	20%		
sum	68 (73.9%)	24 (26.1%)				73 (79.3%)	19 (20.7%)			

*P<0.01. ele, elevation group; non-ele, non-elevation group; red, reduction group; non-red, non-reduction group.

### 3.4 Comparison of baseline clinical characteristics between patients with acute radiation proctitis and those without acute radiation proctitis

Patients were categorized into two groups based on the occurrence of acute radiation proctitis, and their baseline clinical characteristics prior to treatment were compared. Between the two groups of patients, there were no statistically significant differences in terms of age, BMI, and comorbidities (P> 0.05, see **Table 7**).

**Table 7:** Comparison of baseline characteristics between patients with and without scute radiation proctitis.

Items	1-grade acute radiation proctitis (n=24)	0-grade acute radiation proctitis (n=68)	*P*
Age (year)			0.484
	56 (36-73)	55 (35-69)	
BMI (kg/m^2^)			0.411
<18.5	0	0	
18.5-23.9	4 (16.7%)	12 (17.6%)	
24-27.9	6 (25%)	16 (23.5%)	
≥28	14 (58.3%)	40 (58.8%)	
comorbidities			0.877
with	4 (16.7%)	24 (35.3%)	
without	20 (83.3%)	44 (64.7%)	

## 4 Discussion

Previous research has demonstrated the phenotypic alterations of immune cell are influenced by different radiotherapy regimens and different types of tumors. A meta-analysis involving 877 cancer patients indicated (12) a significant reduction in the number of CD3+ T lymphocytes and CD4+ T lymphocytes in peripheral blood following conventional radiotherapy, whereas the count of CD8+ T lymphocytes remained largely unchanged. However, an increase in CD8+ T lymphocytes was observed after stereotactic body radiotherapy (SBRT). Subgroup analyses further revealed that post-radiotherapy phenotypic changes of immune cell vary in different types of tumor. In another study, SBRT was administered for seven patients with advanced lung cancer who were ineligible for surgery (13). Of these, four patients received a dose of 7.5 Gy/fx 8 fractions, while three patients received a dose of 12.5 Gy/fx 8 fractions. Post-treatment, there was a significant increase in natural killer (NK) cells and a decrease in immunosuppressive regulatory T cells, with these changes becoming apparent within 72 hours and persisting for six months. Conversely, some studies suggest that low-dose radiation may enhance NK cell function, whereas high-dose radiation may impair it (14).

In this study, we observed a significant reduction in B cells in postoperative endometrial cancer patients with medium-risk who underwent brachytherapy. This finding suggests that B cells are highly sensitive to radiation, aligning with previous research (15). Additionally, the tumor is excised after surgery, eliminating tumor-associated antigens that would typically trigger a specific immune response. Consequently, a substantial activation of B cells is unlikely (16). B cells are primarily involved in humoral immunity. A reduction in total B cells suggests a weakened humoral immune response, a diminished capacity to combat pathogens, and increased susceptibility to invasion by bacteria, viruses, and other pathogen (17). So it is important to keep a close watch on immune function during brachytherapy.

Beyond the reduction in B cells, our study also identified a notably significant increase in NK cells post-brachytherapy that similar to those observed after stereotactic body radiation therapy (SBRT). This similarity may be attributed to the comparable dose and fractionation schemes of brachytherapy and SBRT. For the first time, we detected an increase in more potent NKT cells following brachytherapy. The anti-cancer efficacy of NKT cells is exponentially greater than that of NK cells, earning them the moniker “special forces”. The observed increase in NKT cells suggests that brachytherapy not only activates NK cells (18) but also stimulates the activation of more powerful NKT cells (19). This finding is highly significant. The observed NK cells and NKT cells in patients of postoperative endometrial cancer accepting brachytherapy suggests an enhancement in the body’s innate immune activation function. This enhancement may facilitate the clearance of potential residual tumor cells and inhibit the proliferation of tumor cells postoperatively. For patients with advanced recurrent and metastatic endometrial cancer, immunotherapy has shown consistent positive outcomes in clinical trials such as GARNET4 (20), KEYNOTE-158 (21) and KEYNOTE-775 (22). Subsequent trials, including RUBY 7 (23), KEYNOTE-868 (24) and AtEnd/ENGOT-en7 (25) have demonstrated that the combination of immunotherapy and chemotherapy exhibits significant efficacy, particularly in patients with deficient mismatch repair (dMMR) or microsatellite instability-high (MSI-H) profiles. In the context of lung cancer, patients with negative programmed death-ligand 1 (PD-L1) expression exhibit poor efficacy when treated with immunotherapy alone. When single lesions are treated with SBRT a dose of 8 Gy/fx 3fractions, the combination of SBRT and immunotherapy significantly improves overall survival (OS) and progression-free survival (PFS) compared to immunotherapy alone (26). The dose fractionation and radiation characteristics of brachytherapy are analogous to those of SBRT. Drawing inspiration from the successful combination of SBRT and immunotherapy in lung cancer, we propose that, for patients with advanced endometrial cancer, particularly those with proficient mismatch repair/microsatellite stable (pMMR/MSS) status, the integration of brachytherapy and immunotherapy may achieve comparable or superior therapeutic outcomes. In addition, the latest researcha shows that radiation can boost anti-tumor immunity by activating interferon (IFN) signaling. The cGAS/STING pathway is crucial in the immune response of cancer cells exposed to radiation. Certain immune cells, like CD8+ T cells and NK cells, showed associations with genes downstream of STING (27, 28). The integration of radiation, STING pathway agonists, and PD-L1 targeted treatments could provide an innovative strategy for treating locally advanced tumors with radio-immunotherapy.

The incidence of adverse reactions associated with brachytherapy is generally low and mild, with no reported cases of grade II or higher acute or chronic radiation cystitis, proctitis, or vaginitis. Specifically, There were 24 cases (26.1%) of grade I acute radiation proctitis and 19 cases (20.7%) of chronic radiation proctitis. Additionally, 14 cases (15.2%) of grade I acute radiation cystitis and 10 cases (10.9%) of chronic radiation cystitis were observed. Furthermore, 18 cases (19.6%) of grade I acute radiation vaginitis and 25 cases (27.2%) of chronic radiation vaginitis were reported. Upon further examination of the relationship between radiation injury and phenotypic changes in immune cells, this study identified that the incidence of acute radiation proctitis (31.9%) was significantly higher in the group with elevated NK cell levels compared to the non-elevated group(8.7%). Furthermore, we also compared the pre-treatment clinical characteristics of patients with and without acute radiation proctitis, noting no statistically significant differences in age, BMI, and comorbidities, and found no significant differences in age, BMI, and comorbidities which verifies the correlation between the phenotypic alterations of immune cells and the occurrence of acute radiation proctitis. The incidence of chronic radiation proctitis in the NK cell elevation group was 26.1%, whereas it was 4.3% in the non-elevation group. Although the difference between these two groups was not statistically significant, the P-value approached 0.05. The proposed mechanism suggests that brachytherapy elicits radiation-induced inflammation within the body, such as acute and chronic radiation proctitis. This inflammatory response is hypothesized to stimulate immune reactions, resulting in phenotypic alterations of immune cells, for instance, an increase in NK cells. An increase in NK cells suggests enhanced non-specific immune function, aiding in inflammation self-repair and reducing inflammation.To ascertain whether other forms of radiation-induced inflammation, such as radiation cystitis and radiation vaginitis, are associated with specific changes in immune cell phenotypes, it is imperative to enhance the sample size in future research endeavors.

In this study, no significant alterations were observed in T cells and their subsets following brachytherapy, including total T cells, cytotoxic T cells, helper T cells, and regulatory T cells. It is well-established that T cells become activated upon encountering specific antigens and subsequently differentiate into various subtypes. In postoperative patients with endometrial cancer, the tumor has been surgically excised, preventing T cell activation by the tumor and thereby resulting in the absence of a specific immune response. Additionally, compared to external irradiation, brachytherapy affects a more localized area, and T cells exhibit moderate sensitivity to radiation. Consequently, no significant changes in T cells and their subsets were detected post-brachytherapy.

While phenotypic NK/NKT expansion suggests innate immune activation, functional studies are needed to confirm enhanced effector activity. Moreover, in this study, blood samples were predominantly collected on the day after radiotherapy or the subsequent day, resulting in a brief interval of blood sample collection.resulting in a brief interval. Continued collection of blood samples at 1, 3, and 6 months post-treatment, coupled with a comprehensive comparison of the phenotypic changes in immune cells before, during, and after brachytherapy, may yield further insights.

## Data Availability

The original contributions presented in the study are included in the article/supplementary material. Further inquiries can be directed to the corresponding author.
